# High Accurate Environmental Sound Classification: Sub-Spectrogram Segmentation versus Temporal-Frequency Attention Mechanism

**DOI:** 10.3390/s21165500

**Published:** 2021-08-16

**Authors:** Tianhao Qiao, Shunqing Zhang, Shan Cao, Shugong Xu

**Affiliations:** Shanghai Institute for Advanced Communication and Data Science, Shanghai University, Shanghai 200444, China; qiaotianhao@shu.edu.cn (T.Q.); cshan@shu.edu.cn (S.C.); shugong@shu.edu.cn (S.X.)

**Keywords:** environmental sound classification, convolutional recurrent neural network, sub-spectrogram segmentation, score level fusion, temporal-frequency attention mechanism

## Abstract

In the important and challenging field of environmental sound classification (ESC), a crucial and even decisive factor is the feature representation ability, which can directly affect the accuracy of classification. Therefore, the classification performance often depends to a large extent on whether the effective representative features can be extracted from the environmental sound. In this paper, we firstly propose a sub-spectrogram segmentation with score level fusion based ESC classification framework, and we adopt the proposed convolutional recurrent neural network (CRNN) for improving the classification accuracy. By evaluating numerous truncation schemes, we numerically figure out the optimal number of sub-spectrograms and the corresponding band ranges, and, on this basis, we propose a joint attention mechanism with temporal and frequency attention mechanisms and use the global attention mechanism when generating the attention map. Finally, the numerical results show that the two frameworks we proposed can achieve 82.1% and 86.4% classification accuracy on the public environmental sound dataset ESC-50, respectively, which is equivalent to more than 13.5% improvement over the traditional baseline scheme.

## 1. Introduction

As a key technology for recognizing and analyzing environmental audio signals, environmental sound classification (ESC) [1] is rapidly developed during the past few years with broad applications in home automation, machine hearing, as well as surveillance. Compared with traditional sound classification tasks, such as music or speech recognition [2], the development of this field is relatively slow. This is due to the reason that ESC requires to cover a wide range of frequency spectrum, non-stationary characteristic, and noise-like fluctuations [3,4,5].

Since traditional ESC methods usually consist of feature extraction and feature-based classification processes, a general extension scheme is to improve the classification accuracy in a block-by-block manner. For example, zero crossing rate, audio tone, and short-time energy have been proposed in [6] to improve the feature extraction capability in the low noise environments, while a significant computational complexity is usually required to achieve a reasonable classification accuracy. To solve this problem, extracting features in frequency domain and using temporal-frequency spectrograms to represent environmental sounds becomes the most widely used method recently [7] on the basis of making full use of the recent developments in the field of image processing tasks. Mel-frequency cepstrum coefficient (MFCC) [8] could be one example, while log mel spectrogram (Logmel) [9] and log Gammatone spectrogram [10] are more popular in the recent years. Another approach is to update the feature-based classification block, and typical examples include K-nearest neighbors [11], random forest [12], support vector machine [13], and Gaussian mixture model [14]. With the recent development of supervised learning technologies, the feature-based classification algorithms have also been extended to cover dictionary learning [4], matrix factorization [15], and deep learning based solutions, such as deep neural networks (DNN) [16].

In recent years, the record of achievable classification accuracy has been updated simply through different combinations of feature extraction methods and feature-based classification blocks [9,17,18,19]. For example, when MFCC is combined with two different DNN structures, multi-layer perception (MLP) and convolutional neural network (CNN), the classification accuracy of 44.9% and 53.1% can be achieved in the public environmental sound dataset ESC-50, and when log Gammatone is used in combination with CNN, the classification accuracy can reach 78.9% in the same dataset. However, the above schemes failed to incorporate some specific domain knowledge and the achievable classification accuracy is in general limited. In addition, we should note that most of the existing researches focus on the improvement of neural networks, which is generally applicable to any machine learning field, but few consider the improved processing method of features specific to environmental sound. As far as we are aware, the following issues need to be addressed at the current stage.

- *Sub-spectrogram segmentation:* It is very necessary to study the spectrograms of environmental sound more carefully. This is because the low frequency spectrum usually contains more fruitful information, as explained in [10]. Although a straight-forward sub-spectrogram segmentation as proposed in [20] is shown to be effective to improve the acoustic scene classification accuracy, the extension to ESC tasks still remains open. In addition, according to the existing literature, the number of sub-spectrogram segments, as well as the truncation rules need to be optimized as well;
- *Attention mechanism:* Another possible approach to improve the ESC performance is to incorporate the attention mechanism like human beings [21,22,23,24,25] in the convolutional feature layers, either through the temporal [24], frequency [26], channel [27] domain information, or even hybrid of them [27]. However, the previous joint attention scheme [27] focuses on combining the temporal and channel knowledge without considering the frequency domain characteristics, and the joint time-frequency feature is not fully exploited. As shown later, with the joint time and frequency domain attention, the ESC accuracy can be greatly improved;
- *Recurrent architecture with data augmentation:* The sound of many consecutive frames, such as helicopter, has strong correlations in the time domain, and the prediction via recurrent architecture will be possible. As shown in [28], exploiting the correlations among different scales of sequences can be applied to improve the classification accuracy as well. However, this method usually requires a large amount of data to support and comes with the problem of limited dataset. Therefore, it is very necessary to jointly consider the effective methods for expanding the dataset, such as mixup [29] and SpecAugment [30].

In this paper, a sub-spectrogram segmentation [31] (Part of this paper has been published in 2019 IEEE International Workshop on Signal Processing Systems) mechanism has been firstly proposed to address the above concerns, which truncates the entire spectrogram into different pieces in order to conduct experiments separately. Score level fusion has been adopted to combine different classification results from different sub-spectrograms. By evaluating numerous truncation schemes, we numerically figure out the optimal number of sub-spectrograms and the corresponding band ranges. Based on that, we propose a joint attention mechanism with temporal and frequency domain to adjust the temporal-frequency feature map, which can be similarly regarded as automatically assigning the weight map to the feature map. Numerical results show that the two frameworks we proposed can achieve 82.1% and 86.4% classification accuracy on ESC-50 dataset, respectively, which is equivalent to more than 13.5% improvement over the traditional baseline scheme.

The rest of this paper contains the following parts. We first gave a brief introduction to log Gammatone spectrogram and different types of DNN in Section 2, and the overviews of our proposed methods are introduced in Section 3. The proposed sub-spectrogram segmentation and temporal-frequency attention mechanism based ESC classification frameworks are, respectively, introduced in Section 4 and Section 5. In addition, we gave a demonstration of the numerical experiments in Section 6 and Section 7 gives a summary of this paper at the end.

## 2. Preliminary

In this section, we give a brief introduction to the well-known log Gammatone spectrogram and different types of DNNs.

### 2.1. Log Gammatone Spectrogram

By performing *T* point discrete short-time Fourier transform (STFT) on the sampled time domain audio signal s(t), the energy spectrum density, ${\left|S(m,n)\right|}^{2}$, can be obtained, and the formula is as follows,

(1) $$\begin{array}{c} {\left|S(m,n)\right|}^{2}={\left|\sum_{t=\frac{nT}{2}+1}^{\frac{(n+2)T}{2}}s\left(t\right)\cdot e^{-\frac{j2\pi mt}{T}}\right|}^{2}, \end{array}$$

for $m\in \left[1,T/2\right]$, $n\in \left[0,N-1\right]$. Then apply *K* order Gammatone-filter banks to it to determine the log Gammatone spectrogram, and the mathematical expression of this process can be expressed as,

(2) $$\begin{array}{c} S_{GT}\left(n,k\right)=\log\left(\sum_{m=1}^{T/2}{\left|S(m,n)\right|}^{2}\cdot H(m,k)\right), \end{array}$$

for $k\in \left[1,K\right]$, where H(m,k) denotes the frequency response of the *k*th Gammatone-filter in the *m*th sub-band. In addition, H(m,k) and the associated time domain impulse response, h(t), can be, respectively, given by,

(3) $$\begin{array}{c} H\left(m,k\right)=\frac{C}{2}\left(k-1\right)!{\left(2\pi b\right)}^{-k}\left[P\left(m\right)+P^{*}\left(-m\right)\right],\\ P\left(m\right)=e^{i\phi }{\left[1+i\frac{\left(m-f_{0}\right)}{b}\right]}^{-k}, \end{array}$$

(4) $$\begin{array}{c} h\left(t\right)=C\cdot t^{k-1}e^{-2\pi bt}cos\left(2\pi f_{0}t+\phi \right),\forall \;t>0, \end{array}$$

where $f_{0}$ and ϕ denote the center frequency and the corresponding phase information, *k* and b>0 denote the order of filter and the decay rate, and *C* is an empirical constant to adjust the entire value.

**Figure 1:**
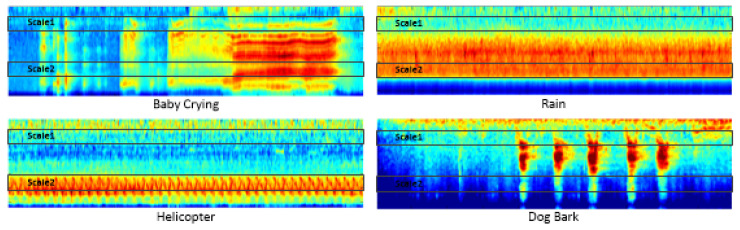
Four typical log Gammatone spectrograms of crying baby, rain, helicopter, and dog bark over the entire frequency band, where the horizontal axis represents time dimension and the vertical axis represents frequency dimension.

In the actual system, usually as shown in [10], select {H(m,k)} to model the characteristics over the entire frequency band, $\left(f_{L},f_{H}\right)$, e.g., from zero to half of the sampling frequency. Moreover, according to [27], the log Gammatone spectrogram is often a two-dimensional channel tensor, which contains itself and its delta information, e.g., $S_{GT}\left(n,k\right)$, and Figure 1 shows the log Gammatone spectrograms of four typical sound classes.

### 2.2. Deep Neural Networks

In general, DNN refers to a more powerful neural network formed by connecting multiple layers of neurons, such as multi-layer perception (MLP), convolutional neural network (CNN), and recurrent neural network (RNN). The design philosophy of MLP and CNN is roughly the same, and the difference is that the neurons within each layer are isolated and neurons across neighboring layers are fully connected in MLP, while the neurons across neighboring layers are connected together through convolution kernels and pooling operations in CNN. In addition, CNN can learn local patterns among different input elements with the help of convolutional architecture, for instance, image pixels or environmental sound spectrograms. RNN is proposed to solve the problem of the temporal correlation among different input vectors or patterns, which is not considered in the above two structures. It cannot only use previous frame-level functions, but also learn complex temporal dynamics. In previous researches, DNN has been proven to be able to handle many challenging tasks in the fields of ESC and computer vision by combining different architectures together.

## 3. Overview of the Proposed High Accurate ESC

**Figure 2:**
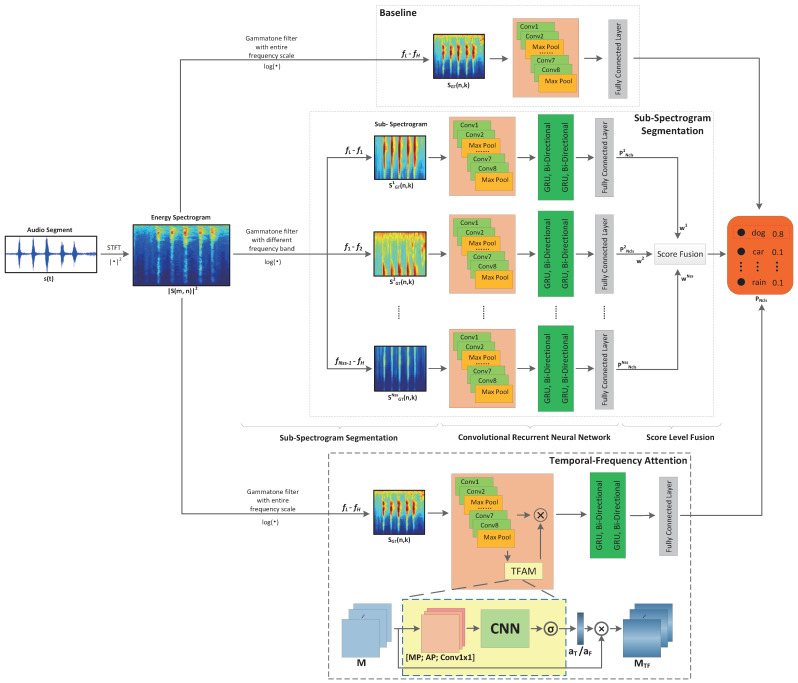
Compare the baseline system, the proposed sub-spectrogram segmentation system and the proposed temporal-frequency attention mechanism system. In this figure, the first branch denotes the baseline system, which extracts log Gammatone spectrogram features on the entire frequency band, the second branch denotes the proposed sub-spectrogram segmentation method, which extracts log Gammatone spectrogram on several sub-frequency bands as illustrated, and the last branch denotes the proposed temporal-frequency attention mechanism (TFAM) and temporal-frequency attention system.

In this part, we put forward two approaches for environmental sound classification, namely *sub-spectrogram segmentation* and *temporal-frequency attention*, where an overview of them is shown in Figure 2.

### 3.1. Overview

In general, the ESC task relies on the observed sound signal s(t) or the equivalent energy spectrum ${\left|S(m,n)\right|}^{2}$ to classify different sound classes. The mathematical expression of the classification task of $N_{cls}$ classes of sounds is,

(5) $$\begin{array}{c} p_{N_{cls}}=F\left(\left\{{\left|S(m,n)\right|}^{2}\right\}\right), \end{array}$$

where $p_{N_{cls}}={\left[p_{1},p_{2},\ldots ,p_{N_{cls}}\right]}^{T}$ denotes the probability distribution across $N_{cls}$ sound classes. The non-linear function F(·) is directly approximated by its equivalent log Gammatone spectrogram and the corresponding neural network defined by θ in the traditional approaches, e.g., $p_{N_{cls}}=G\left(\left\{S_{GT}\left(n,k\right)\right\};\theta \right)$.

### 3.2. Sub-Spectrogram Segmentation

We truncated the whole spectrograms into $N_{ss}$ parts, e.g., $\left(f_{L},f_{1}\right),\ldots ,\left(f_{N_{ss}-1},f_{H}\right)$ instead of generating the log Gammatone spectrogram based on the entire frequency band, and use score level fusion when performing the decision. The overall operations can be described mathematically as follows,

(6) $$\begin{array}{c} p_{N_{cls}}=\sum_{i=1}^{N_{ss}}\omega^{i}p_{N_{cls}}^{i}=\sum_{i=1}^{N_{ss}}\omega^{i}G\left(\left\{S_{GT}^{i}\left(n,k\right)\right\};\theta \right), \end{array}$$

where $p_{N_{cls}}^{i}$ and $\omega^{i}$ denote the score of the *i*th sub-spectrogram and the fusion weight, respectively, and $\sum_{i=1}^{N_{ss}}\omega^{i}=1$. $\left\{S_{GT}^{i}\left(n,k\right)\right\}$ defines the generated log Gammatone spectrograms based on the *i*th band (For illustration purpose, we define $f_{0}=f_{L}$ and $f_{N_{ss}}=f_{H}$.), e.g., from $f_{i-1}$ to $f_{i}$, and G(·;θ) represents a non-linear mapping between log Gammatone spectrogram and classification results.

### 3.3. Temporal-Frequency Attention

The above sub-spectrogram segmentation mechanism only considers the frequency domain, which ignores the temporal domain characteristics. To address this issue, we propose a temporal-frequency attention mechanism (TFAM) as illustrated in Figure 2. Given the input log Gammatone spectrogram, $\left\{S_{GT}\left(n,k\right)\right\}$, we first use CNN to extract temporal-frequency representations. Mathematically, we generate the feature maps, $M\in R^{T\times F\times C}$, on top of the log Gammatone spectrograms according to the following expression,

(7) $$M=H_{1}\left(\left\{S_{GT}\left(n,k\right)\right\};\theta_{1}\right),$$

where *T*, *F* and *C* represents the dimension of feature maps and $H_{1}\left(\cdot ;\theta_{1}\right)$ represents the non-linear transformation provided by CNN.

In order to keep the implementation complexity, we restrict the attention map according to $W_{AT}⊗1$, where $W_{\mathrm{AT}}\in R^{T\times F}$ denotes the temporal-frequency attention patterns, $1\in R^{C}$ denotes an all-one vector with dimension *C*, and ⊗ is Kronecker product as defined in [32]. With the generated attention map, the overall operations can be described as follows,

(8) $$\begin{array}{c} p_{N_{cls}}=G\left(M\cdot \left(W_{AT}⊗1\right);\theta \right), \end{array}$$

where · denotes the element-wise multiplication, and G(·;θ) represents a non-linear mapping between log Gammatone spectrogram and classification results.

## 4. Proposed Sub-Spectrogram Segmentation Based Classification Framework

In this section, we respectively introduce sub-spectrogram segmentation based feature extraction, CRNN based classification, and score level fusion, which are all components of the proposed sub-spectrogram segmentation based classification framework.

### 4.1. Sub-Spectrogram Segmentation

**Figure 3:**
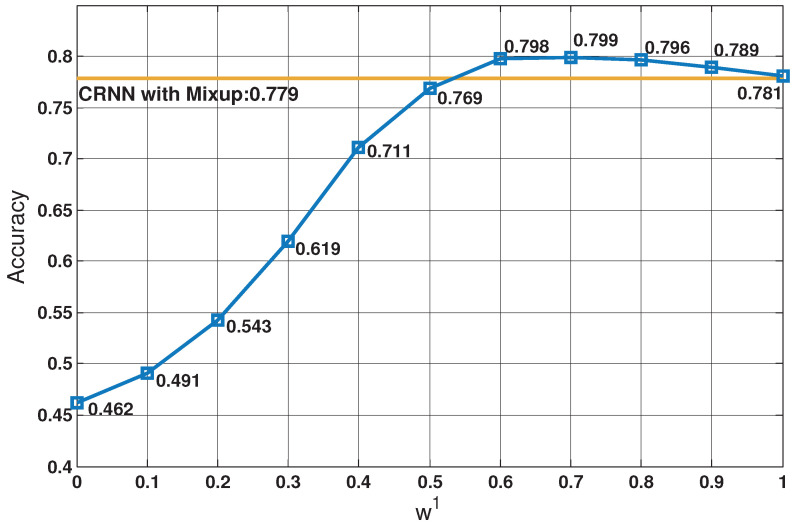
Classification accuracy with different weights. The blue line indicates the classification accuracy of different weights cases, while the orange line indicates the accuracy of CRNN with mixup system.

From Figure 1, we can see that the difference of behaviour in different scales of the spectrogram is really significant. Firstly, we divide the entire log Gammatone spectrogram into two parts, and the parameter settings are $N_{ss}=2$, $f_{L}=0$ kHz, $f_{1}=10$ kHz, and $f_{H}=22.05$ kHz. The classification accuracy on ESC-50 dataset [33] changes with $\omega^{1}$ as shown in Figure 3, and it can be concluded that as long as there is an appropriate weight assignment, the proposed sub-spectrogram segmentation can outperform the baseline system.

Secondly, we identify the optimal number of sub-spectrogram segments through experiments, e.g., $N_{ss}^{⋆}$, and we provide extensive numerical studies. Specifically speaking, we evaluate the system performance under different values of $N_{ss}$ and $\left\{f_{i}\right\}$, and Table 1 lists the results when using the optimal weight coefficients, $\left\{\omega^{i}\right\}$. From Table 1 we can see that the accuracy does *NOT* increase monotonically with regard to $N_{ss}$, and the optimal number of sub-spectrogram segments is $N_{ss}^{⋆}=4$.

### 4.2. CRNN with Mixup

Inspired by the complementary modeling capabilities of CNN and RNN, we combine them into a unified architecture called *convolutional recurrent neural network (CRNN)*, which can be represented as the approximate original non-linear function G(·;θ). The complementary modeling function mentioned here, respectively, refers to using a convolution kernel with a small receptive field on spectrogram features to capture the local spectro-temporal pattern and learning the temporal relationship of the environmental sound features. Specifically, in this system, the learned features obtained through conventional convolutional layer will first be forwarded into the bi-directional gated recurrent unit (GRU) for temporal processing, and then the score of the *i*th sub-spectrogram, $p_{N_{cls}}^{i}$, can be obtained. In addition, detailed architecture of the proposed CRNN and its parameters are presented in Table 2.

For avoiding overfitting that may be caused by the limited training dataset, here we use the data augmentation method, mixup, for constructing virtual training data to achieve the purpose of expanding the training distribution [34]. mixup generates the virtual training data by mixing two training samples, e.g., to attain a mixed virtual feature by mixing a cryingbaby log Gammatone spectrogram and a dogbark log Gammatone spectrogram, the formula can be expressed as

(9) $$\left\{{\tilde{S}}_{GT}^{i}\left(n,k\right)\right\}=\lambda {\left\{S_{GT}^{i}\left(n,k\right)\right\}}_{j}+\left(1-\lambda \right){\left\{S_{GT}^{i}\left(n,k\right)\right\}}_{j^{\prime }},$$

where ${\left\{S_{GT}^{i}\left(n,k\right)\right\}}_{j}$ and ${\left\{S_{GT}^{i}\left(n,k\right)\right\}}_{j^{\prime }}$ are two randomly selected samples in log Gammatone spectrograms of training data. Correspondingly, labels should also be mixed in the same ratio. λ is decided by two hyper-parameters, α and λ∼ Beta(α, α) [34].

Table 3 shows that using CRNN or mixup can increase the classification accuracy by 2.3% and 3.2%, respectively, while CRNN can increase the classification accuracy by 5% higher than the baseline system.

### 4.3. Score Level Fusion

Finally, we experimented to identify the optimal weights, $\left\{\omega^{⋆,i}\right\}$, in score level fusion. We can obtain the optimal weights by exhaustively searching over all possible combinations of $\left\{\omega^{i}\right\}$, and the classification accuracy are shown in Table 4. According to the results, it can be seen that the accuracy of score level fusion can be improved by 2.3% to 3.9% over the uniform weights assignment.

## 5. Proposed Temporal-Frequency Attention Based Classification Framework

In the above method, segmentation boundaries and number of segments need to be optimized over a multi-dimensional search spaces, which is in general computationally prohibitive. In this section, we propose a low-complexity joint temporal-frequency domain searching mechanism to generate the temporal-frequency attention map and figure out a temporal-frequency attention based classification framework with data augmentation. The network structure use here is the same as the CRNN mentioned in Section 4.2.

It is worth mentioning that a similar attention method was introduced in [35]. Apart from the concatenation pattern of the temporal attention and frequency attention, the main difference is the method of obtaining the temporal and frequency attention map. In this paper, we first use the combination of 1 × 1 convolution and pooling to squeeze channel information, while only 1 × 1 convolution is used in [35]. The results in Table 5 are shown that the combination way performs better. Then we use 3 × 3 convolution to learn attention map based on the channel-squeezed feature, while a global average pooling is used in [35]. The learnable attention network usually has ability to learn more valuable information from input feature.

### 5.1. Attention Map Generation

In order to efficiently search the most important temporal-frequency features of an audio spectrogram, we propose a temporal-frequency attention mechanism (TFAM) as shown in Figure 2. Different from the previous sub-spectrogram segmentation based scheme, TFAM directly focuses on the most important frames and frequency bands through training samples, which is more or less the same as semantic segmentation in computer vision tasks. By applying TFAM, the most important temporal-frequency blocks are automatically cached and selected by multiplying an attention map, which eventually helps to the classification tasks thereafter.

To generate the attention map $W_{\mathrm{AT}}$ in (8), we have,

(10) $$W_{\mathrm{AT}}=H_{2}\left(g\left(t,f\right);\theta_{2}\right),$$

where $H_{2}\left(\cdot ;\theta_{2}\right)$ represents the non-linear transformation defined by CNN, and g(t,f) is the concatenated spatial map. Mathematically, g(t,f) can be obtained by the following expression.

(11) $$g\left(t,f\right)=\left\{\begin{array}{c} \begin{array}{cc} \max_{c}\left(M\left(t,f,c\right)\right)⊕ & \max\mathrm{pooling}\\ \frac{1}{C}\sum_{c=0}^{C-1}M(t,f,c)⊕ & \mathrm{average}\mathrm{pooling}\\ {*}_{1\times 1}\left(M\left(t,f,:\right)\right) & 1\times 1\mathrm{convolution} \end{array} \end{array}\right.,$$

where ⊕ denotes the concatenation operation along the channel axis and ${*}_{1\times 1}$ denotes the 1×1 convolution operation. Although we can apply different combinations of pooling and convolution operations, the numerical results in Table 5 show that the concatenated approach achieves better performance in terms of the classification accuracy.

Since the frequency domain characteristics of spectrogram features remains static over different time frames, we choose to process temporal and frequency domains separately as proposed in [36] instead of jointly processing them together as an image. Through this approach, we extract global temporal and frequency attention vectors, e.g., $a_{T}\in R^{T\times 1\times 1}$ and $a_{F}\in R^{1\times F\times 1}$, and generate the final attention map $W_{\mathrm{AT}}$, according to

(12) $$W_{\mathrm{AT}}=a_{T}⊗a_{F}.$$

To obtain $a_{T}$ and $a_{F}$, we forward g(t,f) into a standard CNN network, which consists of three two-dimensional convolutions with 3×3 receptive field for learning the hidden representations and three one-dimensional max pooling layers for reducing the time, frequency or channel dimension. We can use the following formula to describe this process,

(13) $$a_{T}=\sigma \left(H_{3}\left(g\left(t,f\right);\theta_{3}\right)\right),$$

(14) $$a_{F}=\sigma \left(H_{4}\left(g\left(t,f\right);\theta_{4}\right)\right),$$

where $H_{3}\left(\cdot ;\theta_{3}\right)$ and $H_{4}\left(\cdot ;\theta_{4}\right)$ represent the non-linear transformations obtained by CNN, and σ(·) denotes the sigmoid activation function, which is use to restrict the vector elements to a range of (0, 1).

In order to further improve ESC accuracy, we cascade the proposed TFAM blocks after different CRNN pooling layers in Table 2, and the simulation results are shown in Table 6. A classification accuracy up to 83.1% can be reached if the proposed TFAM blocks are cascaded after each CRNN pooling layers, which also outperforms the previous sub-spectrogram segmentation mechanism.

### 5.2. Data Augmentation Schemes

**Figure 4:**
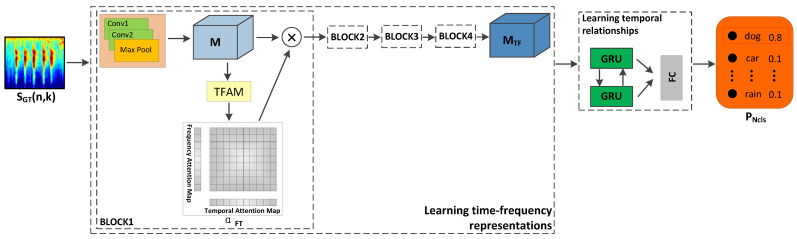
Illustration of our proposed environmental sound classification framework with temporal-frequency attention mechanism (TFAM).

The entire network architecture with CRNN and four TFAM blocks are depicted in Figure 4, where the final learned feature map, $M\cdot \left(W_{AT}⊗1\right)$, is forwarded to bi-directional GRU for the temporal processing. The overall classification results, $p_{N_{cls}}$, are obtained from a fully connected network, with dimension 50×1.

Since we usually have limited sizes of datasets for environmental sound classification, SpecAugment [37] and mixup [34] strategies are adopted to increase the diversity of training sample. SpecAugment applies multiple temporal and frequency masking schemes to generate multiple masked log Gammatone spectrogram, and mixup adopts a randomly mixing strategy between training samples to generate virtual mixed log Gammatone spectrogram and extend the training distribution. By jointly utilizing the above data augmentation schemes, we have the classification accuracy results as listed in Table 7.

## 6. Experiments

In order to prove the effectiveness of our proposed schemes, we numerically perform the experiments on a public environmental sound dataset called “ESC-50” [33] in this section. The ESC-50 dataset collects 2000 environmental recordings, which belong to 50 classes of 5 major categories, including animals, natural soundscapes and water sounds, human non-speech sounds, interior or domestic sounds, and exterior and urban noises. All audio samples are 5 s with a 44.1 kHz sampling frequency. In addition, all the experiments in this paper are obtained through five-fold cross-validation.

### 6.1. Experiment Setup

All the experiments are evaluated on the Nvidia P100 GPU for a fair comparison, and all models are trained by using Keras library with TensorFlow backend. In the training stage, we use the mini-batch stochastic gradient descent with Nesterov momentum of 0.9 and the learning rate scheme of reducing by 10 times per 100 epochs with initial value of 0.1. Moreover, we choose cross entropy as the loss function and the batch size is set as 200. Listed in Table 8 are some other important parameters.

In the following scenarios, a simple CNN architecture shown in Figure 2 is use as a baseline, which models the relation between the log Gammatone spectrogram and the final results. Meanwhile, we numerically compare the classification performance of our proposed schemes with that of the baseline scheme.

### 6.2. Effect of Sub-Spectrogram Segmentation

We analyze the results under the influence of different $N_{ss}$, $\left\{f_{i}\right\}$, and $\left\{\omega^{i}\right\}$, as shown in Table 9. Firstly, we choose some $f_{i}$, and then we can attain a variety of different situations, such as different $N_{ss}$ and the same $N_{ss}$ with different $\left\{f_{i}\right\}$, by combining some of them. Then, we assign different $\left\{\omega^{i}\right\}$ to them and test the classification performance of the models.

The effect of $N_{ss}$ in this system has been analyzed in Section 4.1, and the optimal number is $N_{ss}^{⋆}=4$. Here, we also compare the classification accuracy in three selection ways of $\left\{f_{i}\right\}$, including more segments in low-frequency portion, roughly average segmentation and more segments in high-frequency portion, and Table 9 shows the results, which can prove that a higher classification accuracy can be obtained when more segments are applied in low-frequency portion.

We can reach two conclusions by analyzing the curve in Figure 3, the one is that low-frequency band contains a large proportion of the characteristics of environmental sounds, and the other is that high-frequency band is still indispensable for ESC although it contains few of the characteristics. Therefore, in order to obtain better performance, we appropriately increased $\left\{\omega^{i}\right\}$ of low-frequency segments during fusion, and in Table 9, all $\left\{\omega^{i}\right\}$ are optimal in its corresponding situation.

### 6.3. Accuracy under Sub-Spectrogram Segmentation Based Classification Framework

Further, we compared the classification accuracy in different combinations of mixup, CNN, RNN, segmentation, and score level fusion. As shown in Table 10, the classification accuracy can be improved when using them together. Specifically, our highest classification accuracy is 82.1%, which has an absolutely improvement of 9.2% over the baseline system.

### 6.4. Accuracy under Temporal-Frequency Attention Based Classification Framework

We finally combine different strategies together to improve the overall classification accuracy, including CRNN architecture, different data augmentation schemes, as well as the proposed TFAM blocks. As shown in Table 11, by jointly utilizing all the above strategies, we can achieve a classification accuracy up to 86.4%, which corresponds to 3.9% improvement than sub-spectrogram segmentation based classification framework. This can also significantly demonstrate that the method we proposed is effective.

**Figure 5:**
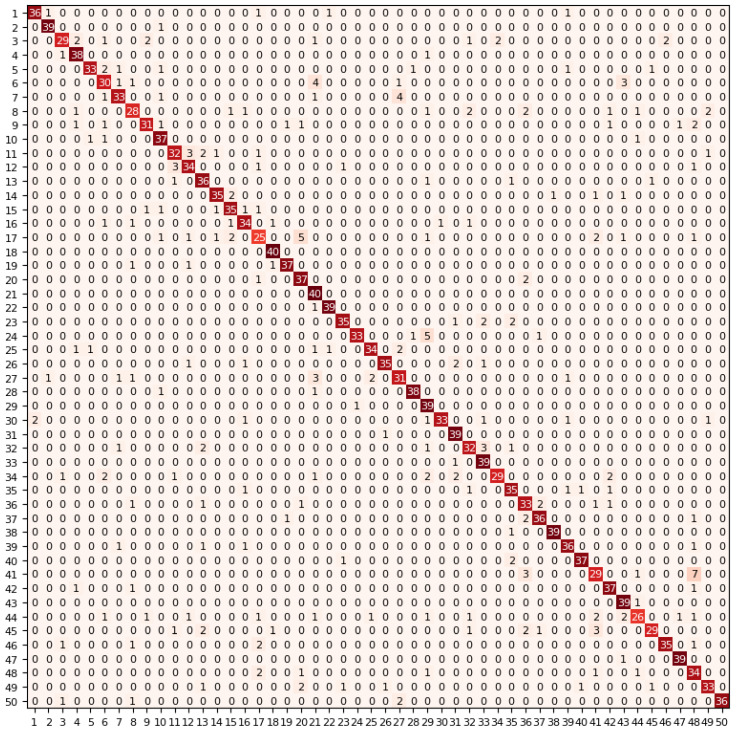
The confusion matrix when the classification accuracy is 86.4%.

In addition, Figure 5 shows the confusion matrix when the classification accuracy is 86.4%. The confusion matrix fully displays the correctness and wrongness of the classification of each class. The horizontal axis and vertical axis in the figure, respectively, represent the predicted labels and the true labels of 50 classes of environmental sound. Among them, the corresponding relationship between the number from 1 to 50 and the environmental sound class is: 1—Dog; 2—Rooster; 3—Pig; 4—Cow; 5—Frog; 6—Cat; 7—Hen; 8—Insect (flying); 9—Sheep; 10—Crow; 11—Rain; 12—Sea waves; 13—Crackling fire; 14—Crickets; 15—Chirping birds; 16—Water drops; 17—Wind; 18—Pouring water; 19—Toilet flush; 20—Thunderstorm; 21—Crying baby; 22—Sneezing; 23—Clapping; 24—Breathing; 25—Coughing; 26—Footsteps; 27—Laughing; 28—Brushing teeth; 29—Snoring; 30—Drinking, sipping; 31—Door knock; 32—Mouse click; 33—Keyboard typing; 34—Door, wood creaks; 35—Can opening; 36—Washing machine; 37—Vacuum cleaner; 38—Clock alarm; 39—Clock tick; 40—Glass breaking; 41—Helicopter; 42—Chainsaw; 43—Siren; 44—Car horn; 45—Engine; 46—Train; 47—Church bells; 48—Airplane; 49—Fireworks; 50—Hand saw.

Finally, we compare the classification accuracy of the method proposed in this paper with the existing methods, as shown in Table 12. It can be seen that compared with most existing methods, the proposed method has obvious advantages in classification accuracy.

## 7. Conclusions

In this paper, we have successively proposed two effective environmental sound classification frameworks based on sub-spectrogram segmentation and temporal-frequency domain attention. The proposed frameworks jointly consider the recurrent network architecture, the data augmentation policies, as well as feature enhancement schemes to improve the classification accuracy of ESC-50. Numerical results show that our proposed frameworks can achieve 82.1% and 86.4% classification accuracy on ESC-50 dataset, respectively, which is equivalent to more than 13.5% improvement over the traditional baseline scheme.

## Figures and Tables

**Table 1:** Classification accuracy under different values of $N_{ss}$.

$N_{\mathrm{ss}}$	$f_{L}$ (kHz)	$\left\{f_{i}\right\}$ (kHz)	$f_{H}$ (kHz)	Accuracy
1	0	-	22.05	77.9%
2	0	10	22.05	79.9%
3	0	6, 10	22.05	81.7%
4	0	3, 6, 10	22.05	82.1%
5	0	3, 6, 10, 15	22.05	81.8%
6	0	3, 6, 10, 13, 16	22.05	81.3%

**Table 2:** Architecture of the proposed convolutional recurrent neural network (CRNN).

Layer	Nums of Filters	Filter Size	Stride	Output Size
Conv1	32	(3, 3)	(1,1)	(128, 128, 32)
Conv2	32	(3, 3)	(1,1)	(128, 128, 32)
Pool1	-	-	(4, 2)	(32,64,32)
Conv3	64	(3, 1)	(1, 1)	(32, 64, 64)
Conv4	64	(3, 1)	(1, 1)	(32, 64, 64)
Pool2	-	-	(2, 1)	(16,64,64)
Conv5	128	(1, 3)	(1, 1)	(16, 64, 128)
Conv6	128	(1, 3)	(1, 1)	(16, 64, 128)
Pool3	-	-	(1, 2)	(16,32,128)
Conv7	256	(3, 3)	(1, 1)	(16, 32, 256)
Conv8	256	(3, 3)	(1, 1)	(16, 32, 256)
Pool4	-	-	(2, 2)	(8, 16, 256)
GRU1	256	-	-	(16, 256)
GRU2	256	-	-	(16, 256)
FC1	nums of classes	-	-	(nums of classes)

**Table 3:** Classification accuracy of whether to use CRNN or mixup.

Network	Mixup	Accuracy
CNN	×	72.9%
CRNN	×	75.2%
CNN	*√*	76.1%
CRNN	*√*	77.9%

**Table 4:** Classification accuracy under different score level fusion strategies.

$N_{\mathrm{ss}}$	$f_{L}$ (kHz)	$\left\{f_{i}\right\}$ (kHz)	$f_{H}$ (kHz)	Fusion	Accuracy
2	0	10	22.05	×	76.2%
2	0	10	22.05	*√*	79.9%
3	0	6, 10	22.05	×	78.1%
3	0	6, 10	22.05	*√*	81.7%
4	0	3, 6, 10	22.05	×	79.8%
4	0	3, 6, 10	22.05	*√*	82.1%
5	0	3, 6, 10, 15	22.05	×	77.9%
5	0	3, 6, 10, 15	22.05	*√*	81.8%

**Table 5:** Classification accuracy comparison for max and average pooling, and 1×1 convolution.

Network	Max Pooling	Avg Pooling	1×1 Conv	Accuracy
CRNN	*√*	×	×	82.0%
CRNN	×	*√*	×	81.8%
CRNN	×	×	*√*	82.1%
CRNN	*√*	*√*	*√*	82.3%

**Table 6:** Classification accuracy comparison for different TFAM allocations.

Network	Layer	Layer	Layer	Layer	Accuracy
	Pool1	Pool2	Pool3	Pool4	
CRNN	*√*	×	×	×	82.3%
CRNN	×	*√*	×	×	82.0%
CRNN	×	×	*√*	×	81.9%
CRNN	×	×	×	*√*	81.9%
CRNN	*√*	*√*	×	×	82.0%
CRNN	*√*	×	*√*	×	82.6%
CRNN	*√*	×	×	*√*	82.0%
CRNN	×	*√*	*√*	×	81.9%
CRNN	×	*√*	×	*√*	82.6%
CRNN	×	×	*√*	*√*	82.1%
CRNN	*√*	*√*	*√*	×	82.2%
CRNN	*√*	*√*	×	*√*	82.0%
CRNN	*√*	×	*√*	*√*	81.6%
CRNN	×	*√*	*√*	*√*	82.7%
CRNN	*√*	*√*	*√*	*√*	83.1%

**Table 7:** Classification accuracy of whether to use mixup or SpecAugment.

Network	Mixup	SpecAugment	TFAM	Accuracy
CRNN	×	×	×	75.2%
CRNN	*√*	×	*√*	83.1%
CRNN	×	*√*	*√*	82.7%
CRNN	*√*	*√*	*√*	86.4%

**Table 8:** Parameter settings in experiments.

Parameters	Definition	Values
$f_{s}$	sampling frequency	44,100
$N_{cls}$	number of classes	50
*T*	STFT point	1024
*N*	frame length	128
*K*	number of Gammatone-filter banks	128
α	Mixup hyper-parameter	0.2

**Table 9:** Classification accuracy under different $N_{ss}$, $\left\{f_{i}\right\}$, and $\left\{\omega^{i}\right\}$.

$N_{\mathrm{ss}}$	$f_{L}$ (kHz)	$\left\{f_{i}\right\}$ (kHz)	$f_{H}$ (kHz)	$\left\{w^{i}\right\}$	Accuracy
1	0	-	22.05	1	77.9%
2	0	10	22.05	0.7, 0.3	79.9%
3	0	10, 20	22.05	0.5, 0.3, 0.2	80.2%
3	0	7, 14	22.05	0.5, 0.2, 0.3	80.6%
3	0	6, 10	22.05	0.5, 0.3, 0.2	81.7%
4	0	10, 15, 20	22.05	0.5, 0.2, 0.2, 0.1	80.3%
4	0	5, 10, 15	22.05	0.4, 0.3, 0.1, 0.2	80.9%
4	0	3, 6, 10	22.05	0.4, 0.2, 0.2, 0.2	82.1%
5	0	10, 13, 16, 19	22.05	0.4, 0.2, 0.1, 0.2, 0.1	81.0%
5	0	5, 10, 15, 20	22.05	0.4, 0.2, 0.1, 0.2, 0.1	80.7%
5	0	3, 6, 10, 15	22.05	0.4, 0.2, 0.2, 0.1, 0.1	81.8%
6	0	3, 6, 10, 13, 16	22.05	0.3, 0.2, 0.2, 0.1, 0.1, 0.1	81.3%
6	0	6, 10, 13, 16, 19	22.05	0.4, 0.1, 0.2, 0.1, 0.1, 0.1	81.2%

**Table 10:** Comparison for different combinations of mixup, CNN, RNN, segmentation, and score level fusion. When using segmentation, the $N_{ss}$, $\left\{f_{i}\right\}$ and $\left\{\omega^{i}\right\}$ are set as 4, {3, 6, 10} and {0.4, 0.2, 0.2, 0.2}, respectively.

Network	Mixup	Segmentation	Fusion	Accuracy
CNN	×	×	×	72.9%
CRNN	×	×	×	75.2%
CNN	*√*	×	×	76.1%
CRNN	*√*	×	×	77.9%
CNN	×	*√*	×	76.1%
CNN	×	*√*	*√*	77.6%
CRNN	×	*√*	×	78.1%
CRNN	×	*√*	*√*	79.5%
CNN	*√*	*√*	×	78.2%
CNN	*√*	*√*	*√*	80.8%
CRNN	*√*	*√*	×	79.8%
CRNN	*√*	*√*	*√*	82.1%

**Table 11:** Comparison for different combinations of CNN, RNN, miuxp, SpecAugment, segmentation, and TFAM.

Network	Mixup	SpecAugment	Segmentation	TFAM	Accuracy
CNN	×	×	×	×	72.9%
CRNN	×	×	×	×	75.2%
CRNN	*√*	×	×	×	77.9%
CRNN	×	*√*	×	×	77.5%
CRNN	×	×	*√*	×	79.5%
CRNN	×	×	×	*√*	80.3%
CRNN	*√*	×	*√*	×	82.1%
CRNN	*√*	×	×	*√*	83.1%
CRNN	×	*√*	×	*√*	82.7%
CRNN	*√*	*√*	×	*√*	86.4%

**Table 12:** Classification accuracy comparison.

Existing Methods	TFAM
PiczakCNN	64.9%
Google Net	67.8%
SoundNet	74.2%
AlexNet	78.7%
WaveMsNet	79.1%
ProCNN	82.8%
Multi-Stream CNN	83.5%
EnvNet-v2	84.9%
ACRNN	86.1%
**TFAM**	86.4%
